# Reduced periprosthetic fracture rate for a cemented anatomical versus a tapered polished stem in hip arthroplasty: A 6‐year follow‐up of a prospective observational cohort study

**DOI:** 10.1002/jeo2.70243

**Published:** 2025-04-16

**Authors:** Anna Josefsson, Michael Axenhus, Raéd Itayem, Sebastian Mukka, Martin Magnéli

**Affiliations:** ^1^ Division of Orthopedics, Department of Clinical Sciences at Danderyd Hospital Karolinska Institutet Stockholm Sweden; ^2^ Department of Orthopaedics, Institute of Clinical Science, Sahlgrenska Academy University of Gothenburg Gothenburg Sweden; ^3^ The Swedish Arthroplasty Register Gothenburg Sweden; ^4^ Department of Orthopaedics Sahlgrenska University Hospital, Region Västra Götaland Gothenburg Sweden; ^5^ Department of Surgical and Perioperative Sciences Umeå University Umeå Sweden

**Keywords:** anatomically shaped stem, hip arthroplasty, periprosthetic femur fracture, polished tapered stem

## Abstract

**Purpose:**

In older patients requiring a hip arthroplasty, the cemented straight collarless polished tapered stems (PTSs) have been linked to an increased risk of periprosthetic femur fractures (PFFs) when compared to anatomically shaped stems (ASs). This study aims to perform a 6‐year follow‐up of PFF rates and other adverse events of an orthopaedic department's full transition from a cemented PTS to a cemented AS.

**Methods:**

A prospective single‐centre cohort study included 1077 patients operated with a cemented hip replacement at Danderyd Hospital, Stockholm, Sweden, between 2012 and 2015. Patients were divided into two groups based on stem design: PTS (*n* = 543) and AS (*n* = 534). Outcomes included the PFF rate, periprosthetic joint infection (PJI) and prosthetic dislocation. A Cox proportional hazards model was used to estimate outcomes.

**Results:**

Most patients (77.2%, mean age 82 years) underwent surgery for a hip fracture. The 6‐year PFF rate was 4.6% for the PTS group and 0.9% for the AS group. PFF patterns differed between groups, with Vancouver B fractures being more common in the PTS group. The AS group had lower rates of PJIs (3.6% vs. 1.7%) and dislocations (4.4% vs. 1.3%) than the PTS group.

**Conclusion:**

Transitioning from a PTS to an AS could reduce the PFF rate and other adverse events in hip arthroplasty. The findings are relevant for hospitals treating older and frail patients, as the mean age in this study was >80 years. Further research in different settings is warranted to confirm these results.

**Level of Evidence:**

Level II.

**Abbreviations**
AS: anatomical stem
ASA: American Society of Anaesthesiologists
CI: confidence interval
CPT: collarless polished tapered (specific stem design)
HA: hemiarthroplasty
HR: hazard ratio
ORIF: open reduction and internal fixation
PFF: periprosthetic femur fracture
PJI: periprosthetic joint infection
PTS: polished tapered stem
THA: total hip arthroplasty

## INTRODUCTION

Despite being introduced as early as 1970, the polished tapered stem (PTS) design of hip arthroplasty remains one of Sweden's most frequently implanted cemented stems [34]. The anatomical stem (AS) is a competing design introduced in 1982. At present, the Lubinus SP2 AS has been the most commonly implanted stem in Sweden for many decades, demonstrating its increasing popularity and widespread adoption [34]. Both types have shown good long‐term results [4, 13, 19, 26, 28]. At our institution, the PTS was the standard cemented stem for decades until the AS replaced it because a growing body of evidence suggests that it was associated with a higher rate of periprosthetic femur fractures (PFFs) [3, 6, 17, 23]. A transition from a PTS to an AS was performed in 2014, and the short‐term results of the change in routines have been published [21]. The PFF rate dropped from 3.3% to 0.4% with the transition from a PTS to an AS [21].

Recent international literature highlights the evolving understanding of cemented stem designs and their impact on clinical outcomes. The review by Giebel et al. emphasizes the importance of selecting cemented stems based on patient‐specific factors such as bone quality, activity level, and femoral geometry [11]. This aligns with findings from various national joint registries, including the National Joint Registry for the United Kingdom and the Australian Orthopaedic Association National Joint Replacement Registry [24], which report differing trends in the adoption of PTS and AS designs based on patient demographics and surgical preferences. Although PTS has demonstrated excellent long‐term survivorship, increasing evidence suggests that ASs may provide improved fracture resistance, particularly in frail populations. However, despite short‐term studies supporting the benefits of AS, there remains limited knowledge on how fracture risk evolves over extended follow‐up periods. Long‐term complications such as aseptic loosening, late‐onset periprosthetic fractures, and implant survival beyond the initial years remain underexplored, necessitating studies with longer observation periods to fully assess the durability and safety of AS implants in clinical practice.

PFFs are severe and challenging complications after hip arthroplasty, often leading to significant morbidity and reduced quality of life [18]. These fractures have been linked to an increased mortality rate, especially in older and frail patient populations [7, 32] and are associated with increased healthcare costs [31].

This study aims to perform a 6‐year follow‐up of PFF rates and other adverse events after a change from a cemented PTS to a cemented AS.

## MATERIALS AND METHODS

### Study design, setting and participants

This prospective observational single‐centre cohort study included patients operated on with a cemented stem at the Orthopaedic Department of Danderyd Hospital with either a PTS (from November 2011 to December 2013) or an AS (November 2013 to December 2015). Danderyd Hospital, Stockholm, Sweden, is a university hospital affiliated with Karolinska Institutet, serving approximately 500,000 inhabitants. This prospective cohort study includes all primary hip arthroplasty operations performed at the Orthopaedic Department, Danderyd Hospital. Only cemented stems were included, and uncemented and modular stems were excluded. The standard cemented stem for hemiarthroplasty (HA) and total hip arthroplasty (THA) operations was a PTS (CPT, Zimmer Inc.) until January 2014. After a policy change, the PTS was replaced by an AS (Lubinus SP2, Waldemar Link), introduced in November 2013. November and December 2013 served as a training period for the new AS stem. Both stems were accessible until December 2013; after January 2014, only the AS stem was available. The study thus includes the 2 months of coexisting stems, the training period, and the learning curve for AS in HA and THA. If a patient underwent bilateral operations, only the first hip was included in the analysis. An ethical application for this study was approved by the local ethical review board. For this observational study, the need for informed consent was waived by the board (DNR:213/285‐31/2).

### Exposure

Both stems were used for HA and THA. We included acute (hip fractures) and elective procedures (degenerative joint disease). Surgery was performed by a consultant orthopaedic surgeon or by a registrar under the supervision of a consultant. A direct lateral (Gammer) approach was used for hip fractures [10]. A posterolateral approach (Moore) was mostly used for elective surgery. Cemented and uncemented cups, including dual mobility cups, were used. Head sizes for the THAs ranged from 22 to 36 mm in diameter. For the HA, a unipolar CoCr head was employed.

### Variables

We collected information on the date of operation and reoperation, stem type, cup type (for THA), age, sex, cognitive dysfunction (yes/no, classified by the treating surgeon; temporary confusion was not considered cognitive dysfunction), American Society of Anaesthesiologists (ASA) score [8], indication for surgery, type of arthroplasty (HA or THA), and surgical approach. Periprosthetic fractures were classified according to the Vancouver system [2] by a senior consultant orthopaedic surgeon. Clinical and radiographic outcomes for patients with a PPF were evaluated based on a 5‐point scale: (1) Good outcome (healed without apparent sequelae), (2) Intermediate outcome (healed with impairment to walking ability), (3) Poor outcome (major limitation/did not heal), (4) Further surgery needed and (5) Deceased as a direct consequence of hip complication.

### Data sources

We used the unique personal identification numbers to identify patients. Data were collected prospectively throughout the study by searching our in‐hospital surgical and medical databases. A digital case report form was used during the study period. The Swedish Arthroplasty Register was used to identify participating hips at baseline and any reoperations performed outside our hospital (no such events were found). All patients were followed for a period of 6 years from the date of their primary surgery, and only events occurring within this timeframe were included in the study.

### Sample size

A power analysis conducted before the study started showed that a minimum of 431 patients in each group would provide a significance level of 5% and a power of 80% to detect a statistically significant difference in the PFF rate with the assumption of a 3% PFF rate in the PTS group and 0.5% rate in the AS group. Because the orthopaedic department then performed 250–300 cemented hip arthroplasties per year, the sample size was achieved by including all patients operated on 2 years before and 2 years after the implant change.

### Statistical methods

We tested for differences in hip fracture proportion between the PTS and AS groups with a two‐sided *Z* test. We used a Cox proportional hazards model for the outcomes. Follow‐up time was defined as time to death, reoperation (for periprosthetic joint infection [PJI], PFF or other revision) or a maximum follow‐up of 6 years. We adjusted for the following exposure variables: type of stem (PTS or AS), age, sex, body mass index, ASA category (1 and 2 or 3 and 4), cognitive dysfunction, indication (fracture or degenerative joint disease), and surgical approach. The results are presented as hazard ratios (HRs) with 95% confidence intervals (CIs). The proportional hazards assumption was tested with the Grambsch and Therneau analysis on scaled Schoenfeld residuals. All statistical computations were performed using R version 4.2.2.

## RESULTS

### Study subjects and descriptive data

A total of 2005 hip arthroplasties were performed during the study period. After excluding uncemented stems, the study cohort comprised 1117 operations in 1076 patients. Of these 1076 patients, 41 had bilateral procedures; however, only the first operation was included in the study (during the work with this publication, an error in the previous publication on the 2‐year results, an error that one patient with bilateral surgeries was included, was found). The two groups were similar in size: PTS, *n* = 542 (50.4%) and AS, *n* = 534 (49.6%). Hip fracture was the most common indication for surgery (76.9%, *n* = 827), and the proportion of hip fracture patients differed between the groups: PTS 79.7% (*n* = 432) and AS 74% (*n* = 395) (*p* = 0.031). The groups had no major differences in baseline demographics (Table 1). The 6‐year mortality in the cohort was 56.4% (*N* = 607), with no significant difference between the two groups: PTS, 57.2% (*n* = 310) and AS, 55.6% (*n* = 297) (HR: 0.9, 95% CI: 0.8–1.1).

**Table 1:** Baseline demographics between groups.

	Fracture		Degenerative		Overall	
	PTS (*N* = 432)	AS (*N* = 395)	PTS (*N* = 110)	AS (*N* = 139)	PTS (*N* = 542)	AS (*N* = 534)
Sex						
Male	124 (28.7%)	109 (27.6%)	32 (29.1%)	27 (19.4%)	156 (28.8%)	136 (25.5%)
Female	308 (71.3%)	286 (72.4%)	78 (70.9%)	112 (80.6%)	386 (71.2%)	398 (74.5%)
Age						
Mean (SD)	82.2 (8.57)	82.2 (8.18)	79.1 (6.89)	79.9 (7.13)	81.5 (8.35)	81.6 (7.99)
ASA class						
1–2	87 (20.1%)	110 (27.8%)	49 (44.5%)	76 (54.7%)	136 (25.1%)	186 (34.8%)
3–4	345 (79.9%)	285 (72.2%)	61 (55.5%)	63 (45.3%)	406 (74.9%)	348 (65.2%)
Body mass index						
Mean (SD)	23.9 (3.83)	23.5 (4.31)	26.1 (4.84)	26.0 (4.70)	24.3 (4.16)	24.1 (4.55)
Cognitive dysfunction						
No	276 (63.9%)	260 (65.8%)	107 (97.3%)	132 (95.0%)	383 (70.7%)	392 (73.4%)
Yes	156 (36.1%)	135 (34.2%)	3 (2.7%)	7 (5.0%)	159 (29.3%)	142 (26.6%)
Type of arthroplasty						
Total hip arthroplasty	99 (22.9%)	110 (27.8%)	110 (100%)	139 (100%)	209 (38.6%)	249 (46.6%)
Hemiarthroplasty	333 (77.1%)	285 (72.2%)	0 (0%)	0 (0%)	333 (61.4%)	285 (53.4%)
Surgical approach						
Direct lateral	404 (93.5%)	349 (88.4%)	7 (6.4%)	6 (4.3%)	411 (75.8%)	355 (66.5%)
Posterolateral	28 (6.5%)	46 (11.6%)	103 (93.6%)	133 (95.7%)	131 (24.2%)	179 (33.5%)

Abbreviations: AS, anatomical stem; ASA, American Society of Anaesthesiologists; PTS, polished tapered stem; SD, standard deviation.

### Reoperation caused by adverse events

The reoperation rate due to adverse events was 6.7% (*n* = 72), with 9.4% (*n* = 51) in the PTS group and 3.9% (*n* = 21) in the AS group. Of these patients, 23 had more than one reoperation, with 1 undergoing eight reoperations (seven for dislocation and one for a PFF). Patients with hip fractures had a reoperation rate of 7.4% (*n* = 61) compared to 4.4% in patients with degenerative joint disease (*n* = 11) (*p* = 0.135).

#### PFFs

**Figure 1:**
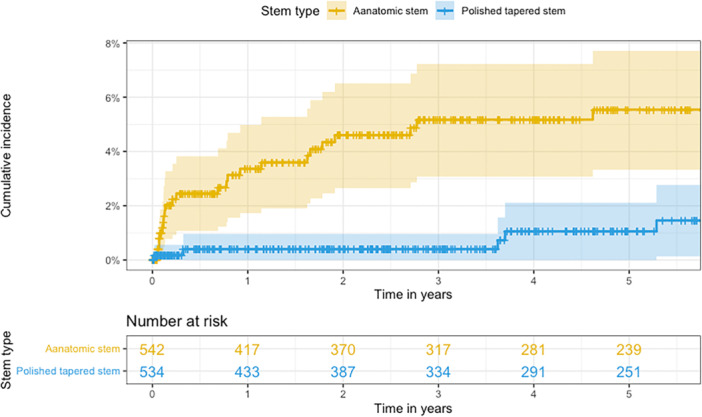
Cumulative incidence curves for periprosthetic fractures. AS in yellow and PTS in blue. AS, anatomical stem; PTS, polished tapered stem.

Within 6 years of operation, 28 (2.7%) patients sustained a PFF. The PPF rate was higher in the PTS group (4.6%, *n* = 24) compared to the AS group (0.9%, *n* = 5) (Table 2 and Figure 1).

**Table 2:** Adverse events in both groups.

	Fracture		Degenerative		Overall	
	PTS (*N* = 407)	AS (*N* = 395)	PTS (*N* = 98)	AS (*N* = 139)	PTS (*N* = 505)	AS (*N* = 534)
Periprosthetic fracture						
	20 (4.9%)	4 (1.0%)	3 (3.1%)	1 (0.7%)	23 (4.6%)	5 (0.9%)
Periprosthetic joint infection						
	15 (3.7%)	7 (1.8%)	3 (3.1%)	2 (1.4%)	18 (3.6%)	9 (1.7%)
Dislocation						
	17 (4.2%)	7 (1.8%)	5 (5.1%)	0 (0%)	22 (4.4%)	7 (1.3%)
Vancouver classification						
A	2 (10.0%)	1 (25.0%)	0 (0%)	0 (0%)	2 (8.7%)	1 (20.0%)
B1	4 (20.0%)	0 (0%)	1 (33.3%)	0 (0%)	5 (21.7%)	0 (0%)
B2	12 (60.0%)	0 (0%)	2 (66.7%)	0 (0%)	14 (60.9%)	0 (0%)
B3	1 (5.0%)	0 (0%)	0 (0%)	0 (0%)	1 (4.3%)	0 (0%)
C	1 (5.0%)	3 (75.0%)	0 (0%)	1 (100%)	1 (4.3%)	4 (80.0%)

Abbreviations: AS, anatomical stem; PTS, polished tapered stem.

**Figure 2:**
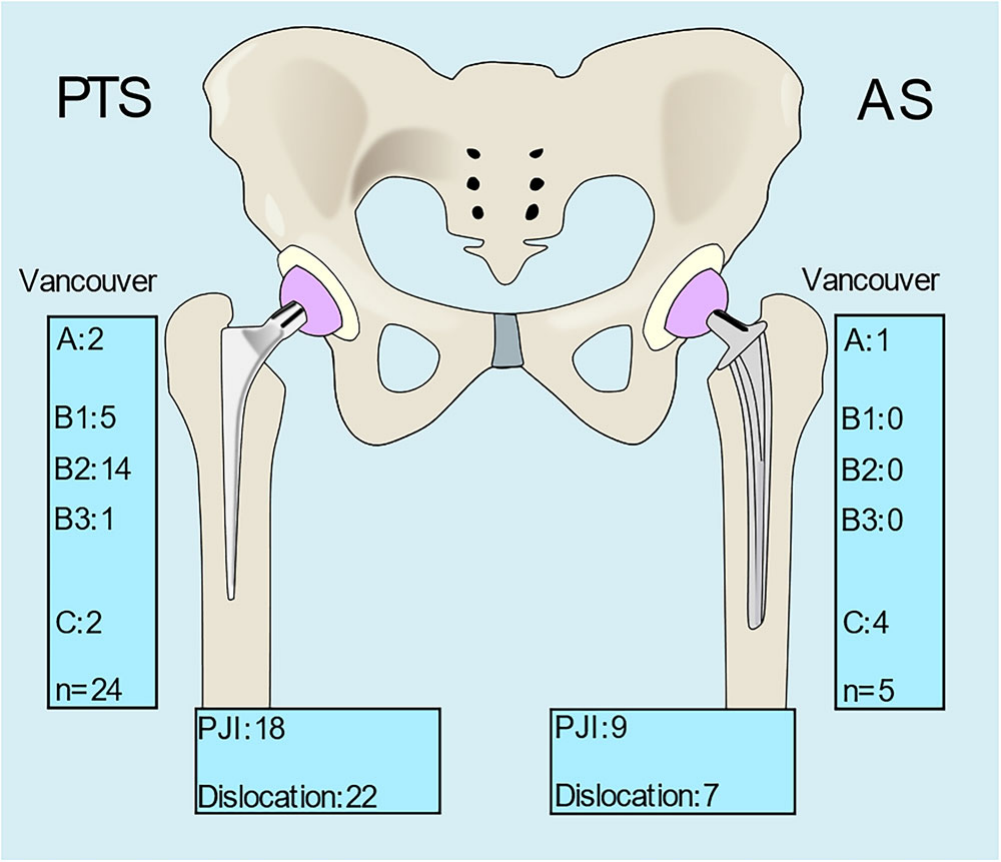
Summary of adverse events in the polished tapered stem (PTS) and anatomical stem (AS) groups. PJI, periprosthetic joint infection.

A PFF was more common in hip fracture patients (3.0%, *n* = 25) compared to degenerative joint disease patients (1.6%, *n* = 4). The median time from operation to a PFF was 0.8 years (mean: 1.3 years). The fracture pattern of PFFs differed between the two groups (Figure 2).

PFFs in the PTS group included all Vancouver classes, with B2 being the most common. In contrast, PFFs in the AS group were all Vancouver A and C fractures. Nine of the 24 PFFs in the PTS group were treated with fracture fixation and 15 with revision arthroplasty. All PFFs in the AS group were treated with fracture fixation. Two Vancouver A fractures were treated with fracture fixation and one with stem revision. All Vancouver B1 and C fractures were treated with open reduction and internal fixation (ORIF). One Vancouver B2 and B3 fracture was treated with ORIF, and 13 with stem revision. The clinical outcome for PFFs in the PTS group was good for 12 patients, intermediate for 7 and poor for 3. Two patients required further surgery. In comparison, outcomes for the AS group were good for four and intermediate for one.

#### PJIs

Within 6 years after surgery, 27 (2.5%) patients contracted a PJI. The incidence of PJIs was 3.6% (*n* = 18) in the PTS group and 1.7% (*n* = 9) in the AS group (*p* = 0.129) (Table 2).

The PJI rate in hip fracture patients was 2.7% (*n* = 22) compared to 2% (*n* = 5) in degenerative joint disease patients (*p* = 0.730). The median time from primary surgery to PJI was 23 days (mean: 3.9 months).

#### Prosthetic dislocation

Some 29 (2.9%) patients experienced at least one dislocation within 6 years after primary surgery. The dislocation rate was higher in the PTS group (4.4%, *n* = 22) than in the AS group (1.3%, *n* = 7) (*p* = 0.0041). For hip fracture patients, the dislocation rate was 3.1% (*n* = 26) compared to degenerative joint disease patients (2%, *n* = 5) (*p* = 0.470). The median time from primary surgery to dislocation was 21 days (mean: 1.6 months). HA patients (all with fracture as an indication for primary surgery) had a dislocation rate of 3.4% (*n* = 21) and THA patients 2.3% (*n* = 10) (*p* = 0.321).

#### Risk factors

Female sex was associated with a lower risk of events compared to males, with an adjusted HR of 0.5 (95% CI: 0.2–1.0). Patients classified as ASA 3–4 had a higher risk compared to ASA 1–2, with an adjusted HR of 2.7 (95% CI: 0.9–8.1). Patients with AS‐type stems had a significantly lower risk of events compared to those with PTS‐type stems, with an adjusted HR of 0.2 (95% CI: 0.1–0.6) (Table 3).

**Table 3:** Crude and adjusted hazard ratios (HRs).

			Crude		Adjusted	
Variable	Total	Event	HR	2.5%–97.5%	HR	2.5%–97.5%
Age	81.6 (±8.2)	81.0 (±7.7)	1.0	1.0–1.0	1.0	0.9–1.0
Body mass index	24.2 (±4.4)	23.1 (±3.6)	0.9	0.8–1.0	0.9	0.8–1.0
Sex						
Male	292	12 (4.1%)	1.0	ref	1.0	ref
Female	784	17 (2.2%)	0.4	0.2–0.9	0.5	0.2–1.0
ASA class						
1–2	322	4 (1.2%)	1.0	ref	1.0	ref
3–4	754	25 (3.3%)	3.4	1.2–9.9	2.7	0.9–8.1
Cognitive dysfunction						
No	775	22 (2.8%)	1.0	ref	1.0	ref
Yes	301	7 (2.3%)	1.1	0.5–2.6	0.8	0.3–2.0
Indication for surgery						
Fracture	827	25 (3.0%)	1.0	ref	1.0	ref
Degenerative	249	4 (1.6%)	0.4	0.1–1.2	0.6	0.2–1.8
Type of stem						
PTS	542	24 (4.4%)	1.0	ref	1.0	ref
AS	534	5 (0.9%)	0.2	0.1–0.5	0.2	0.1–0.6

Abbreviations: AS, anatomical stem; ASA, American Society of Anaesthesiologists; PTS, polished tapered stem.

## DISCUSSION

This prospective observational follow‐up study on transitioning practice from a PTS to an AS for cemented hip arthroplasty reduced the PFF rate [11]. Our findings revealed that the 6‐year, PFF rate was lower in the AS group than in the PTS group (0.9% vs 4.4%). Moreover, the periprosthetic fracture morphology differed, with the AS group only experiencing Vancouver A and C fractures while the PTS group primarily sustained Vancouver B fractures. Our findings are relevant for hospitals treating older and frail patients.

These findings align with Giebel et al. [11], who emphasize the importance of stem type and fixation technique in minimizing complications. Additionally, Morlock et al. [22] highlight the impact of implant choice on revision rates and mortality, reinforcing our findings that AS stems may contribute to improved long‐term outcomes.

Our findings build upon previously published short‐term results, which demonstrated a significant reduction in PFF rates within the first 2 years following the transition from PTS to AS. In the current extended follow‐up of 2–6 years, we continue to observe a lower overall incidence of fractures in the AS group. However, it is noteworthy that in the AS group, three out of five fractures occurred after 3.5 years of follow‐up. This contrasts with the earlier occurrence of fractures in the PTS group, suggesting potential differences in failure mechanisms between the two stem designs.

These findings highlight the importance of long‐term monitoring, as the underlying mechanisms contributing to late periprosthetic fractures with AS could relate to factors such as progressive osteopenia, altered load transfer, and the natural ageing process of the bone‐cement interface. Further research is needed to explore whether additional preventive measures, such as optimized stem alignment and enhanced cementing techniques, could mitigate the risk of late fractures with AS.

The fixation principles of PTS and AS designs are different and may affect the timing of PPF: PTSs subside inside the cement mantle to achieve an even load bearing, and the ASs are fixed in the cement mantle. The wedge‐shaped design renders PTSs more likely to result in metaphyseal split fractures than ASs [5, 25]. In our study, Vancouver B fractures occurred in 20 out of 24 PTSs, while none occurred in AS revisions. Higher rates of PFFs have been observed in frail hip fracture populations with PTS compared to AS [15, 17, 23]. The stem size chosen in PTS has been proposed to modify the risk of a PFF, but this remains to be examined in larger studies and is not within the scope of this study [16]. Furthermore, a recent study by Girardot et al. shows that there is no difference in complication rates between conventional and shortened stems, highlighting the need to identify other stem characteristics that might contribute to increased complication rates [12]. ASs, which rely on a shape‐closed fixation with a more anatomical fit and reduced micromotion, may initially provide greater stability compared to the subsidence‐based force‐closed PTS design. The results from this study suggest that AS may delay the onset of fractures that would have otherwise occurred earlier with PTS but may not eliminate the long‐term risk entirely. The biomechanical explanation of why needs to be studied further.

There are other differences between the specific stems (CPT and Lubinus SP2) used in this study. CPT has been proposed to overcorrect offset because of design features and an increased risk of varus alignment. Restoring offset could be an important factor in preventing dislocation [9, 15]. Our study found no differences in dislocation rates between stem types.

PJIs were included as an outcome to ensure that the transition to a new stem did not increase other adverse events. PJIs are associated with extended surgical time, which could be expected when introducing a new implant [29]. Surprisingly, the AS group exhibited a lower PJI rate (1.7% vs. 3.3%), but this difference was nonsignificant. Despite the fact that the introduction of AS also includes a learning curve, we did not find an increase in surgeon‐related adverse events during the study period.

The choice between fixation and revision arthroplasty for a PFF, especially near a PTS, remains under discussion [14, 29, 30]. Recently, revision arthroplasty has been linked to higher reoperation rates, longer surgical waiting times, higher transfusion rates and the need for critical care compared to fixation [14, 29]. The choice of cemented or uncemented revision stems does not seem to alter the reoperation rates [29, 33]. Two patients required reoperations after initial treatment of the PFFs. By minimizing the incidence of complex fractures requiring revision, AS stems can contribute to substantial cost savings for healthcare systems, particularly in older, frail populations who are at greater risk of complications. Furthermore, reducing reoperation rates and associated morbidity can lead to improved patient outcomes, fewer hospital readmissions, and shorter overall recovery times, enhancing both cost efficiency and patient quality of life. Future studies should quantify these economic benefits by integrating clinical outcomes with cost analyses, further validating the cost‐effectiveness of ASs in hip arthroplasty.

While the two groups showed similar baseline demographics, a slight difference was noted in the percentage of hip fracture patients in the PTS group (80% vs. 74%). Given their high risk of mortality and adverse events [20], the PTS group may inherently have a higher risk of adverse events. However, the AS group was comprised predominantly of hip fracture patients. For PFFs, we adjusted for fragility factors (e.g., ASA score, cognitive dysfunction and hip fracture) in the Cox regression and could not identify any other significant factors apart from stem type. These findings contrast with previously reported risk factors for PFFs [27].

The strengths of this study include its prospective design and consecutive inclusion of patients. Another strength is the completeness of follow‐up data on reoperations and cross‐reference with a national quality register for identifying potential reoperations performed elsewhere. The follow‐up time in our study, considered mid‐term by Ahmad et al., is still too short to fully assess very long‐term outcomes, such as aseptic stem loosening, late‐onset periprosthetic fractures, and other potential complications that may arise a decade or more post‐surgery [1]. Aseptic loosening, for example, is often a gradual process influenced by factors such as implant design, cement technique, and patient activity levels, and it typically becomes more apparent over longer time horizons. Similarly, later periprosthetic fractures may emerge due to changes in bone quality, ongoing stress at the cement‐bone interface, or cumulative wear‐related damage.

This limitation underscores the need for extended follow‐up periods in future studies to better capture these long‐term complications. Furthermore, while our study provides valuable insights into the transition from PTS to AS stems, it is based on a single‐centre cohort. Multi‐centre trials with larger and more diverse patient populations are essential to validate our findings and evaluate whether the observed benefits of ASs are consistent across different healthcare systems and surgical settings.

A limitation of this study is the chronological nature of the transition from PTS to AS, which could introduce a potential learning curve bias. Although only one patient received an AS during the 2‐month concurrent period—limiting the possibility of a direct comparison during this phase—the overall observed reduction in fracture rates may still reflect both improvements in implant design and evolving surgical proficiency over time.

Even though 6 years could be classed as mid‐term, it is relatively long in this cohort, given that most patients suffered from hip fractures and their mean age was nearly 82 years. The follow‐up time is, however, too short to explore later complications, such as aseptic stem loosening and later periprosthetic fractures. The mortality rate following a hip fracture in Sweden can reach up to 20% within 4 months after the event [18]. However, the total study cohort is unbalanced, given the large proportion of hip fracture patients. Furthermore, there was an imbalance in the proportion of hip fracture patients in the two groups, with more hip fracture patients in the PTS group. Including elective and acute patients in a hip arthroplasty cohort can be risky as they are two distinct patient groups. Still, we included both groups and separately presented the results for elective primary and hip arthroplasty for hip fractures. Other limitations of this study are the single‐centre design and the lack of randomization for stem type. We used Cox regression to adjust for potential confounding variables, but the risk of residual confounding is evident. The prospective study design and follow‐up time counterbalanced the limited sample size.

## CONCLUSION

Our study concludes that an orthopaedic department can reduce the PFF rate by transitioning from a PTS to an AS for cemented hip arthroplasty. This conclusion is based on an older and frail patient population that includes nearly 80% of hip fracture patients. In addition to the lower PFF incidence, the AS group only experienced Vancouver A and C fractures. Thus, the AS group could be treated with fracture fixation instead of the more complex revision arthroplasty used for Vancouver B2 and B3 fractures.

These findings underscore the importance of tailoring implant selection to patient‐specific factors, such as age and bone quality, to improve outcomes. However, longer follow‐up studies are necessary to assess the durability of these results and monitor for late complications. Furthermore, multi‐centre trials would help validate the generalizability of these findings across different healthcare settings and patient populations, providing a more comprehensive understanding of the long‐term benefits and challenges associated with ASs.

## AUTHOR CONTRIBUTIONS

All authors contributed to the study conception and design. Data collection and analysis were performed by Martin Magnéli and Sebastian Mukka. The first draft of the manuscript was written by Martin Magnéli, Anna Josefsson and Raéd Itayem, and all authors commented on previous versions of the manuscript. All authors read and approved the final manuscript.

## CONFLICT OF INTEREST STATEMENT

The authors declare no conflicts of interest.

## ETHICS STATEMENT

The study was conducted in accordance with the ethical principles of the Helsinki Declaration and was approved by the Ethics Committee of Karolinska Institutet (dnr 2013/285‐31/2). According to the ethical permission, individual consent was not needed from the patients in this observational cohort.

## Data Availability

The data set used and analyzed during the current study are available from the corresponding author on request.
